# Construction of a prognostic risk model for uveal melanoma based on immune-related long noncoding RNA

**DOI:** 10.1097/MD.0000000000039385

**Published:** 2024-09-06

**Authors:** Nengqi Lin, Ruohan Lv, Dongliang Yang, Wei Liu

**Affiliations:** a Department of Ophthalmology, Union Hospital, Tongji Medical College, Huazhong University of Science and Technology, Wuhan, China; b The First Clinical College, Tongji Medical College, Huazhong University of Science and Technology, Wuhan, China; c Department of General Education Courses, Cangzhou Medical College, Hebei, China.

**Keywords:** immune-related, lncRNA, prognostic model, uveal melanoma

## Abstract

Uveal melanoma (UM) is a common health challenge worldwide as a prevalent intraocular malignancy because of its high mortality rate. However, clinical workers do not have an accurate prognostic tool now. Immune function is closely related to tumor development. Interestingly, researchers have identified that long noncoding RNAs (lncRNAs) are tightly associated with biological processes at the cellular level, particularly their involvements in immune response and its regulation of the growth of tumor cells. Hence, lncRNAs may be involved in the progression of uveal melanoma. UM patients’ RNA expression matrices were extracted from TCGA database. The targeted immune genes were filtered by weighted correlation network analysis and the immune-related lncRNAs with a high prognostic relevance were obtained by Cox regression analysis and least absolute shrinkage and selection operator regression analysis. Each sample was scored according to those lncRNA expression and divided into high-risk and low-risk group. We confirmed the sensitivity and independence of our risk model compared to the tumor mutation burden score. Finally, we demonstrated the clinical relevance of our model by examining its sensitivity to different drugs. The risk score based on our risk model was significantly independent of other clinical parameters in either univariate (hazard ratio = 109.852 [15.738–766.749], *P* value < .001) or multivariate (hazard ratio = 114.075 [15.207–855.735], *P* value < .001) analyses. The ROC curves of this model imply high predictive accuracy for 1-year, 3-year, and 5-year survival (1-year area under the curve [AUC] = 0.849, 3-years AUC = 0.848, and 5-years AUC = 0.761). Our study revealed that immune-related lncRNAs are significant in the clinical diagnosis, treatment and prognosis of UM patients. We successfully constructed a lncRNA-based prognostic risk model which may serve as a future reference for the diagnosis and prognosis of UM. Based on this model we also validated the sensitivity of some cancer drugs, which has implications for the future immunotherapy and drug development.

## 1. Introduction

In ophthalmic diseases, uveal melanoma (UM) is of great invasion as a highly prevalent malignancy.^[1]^ Consisting of iris, choroid and ciliary body, the uvea is a mixed layer of blood vessels and nerves located in the middle of the eyeball wall. Statistically, the incidence of UM is 4% in the iris and 6% in the ciliary body, while it is as high as 90% in the choroid.^[2]^ UM patients have a poor prognosis for survival due to the high malignancy of the tumor. A more recent cohort study of 10,678 samples found the possible lifetime risk of death was 40% to 43%.^[3]^ However, the lack of the effective prognostic predictor for UM still exists. It has been reported that the progression of UM is strongly associated with G protein-coupled receptor-mediated signaling activation processes, with extensive mutations in genes such as GNAQ, GNA11, and BAP1 in primary UM patients.^[4–6]^ However, no reports have pointed to a prognostic significance of these mutations. A specific immune segregation (also known as immune privilege) exists in eyes. The ocular microenvironment induces Treg cell production capable of reducing damage to the eye by cellular infiltration in inflammatory cells, while its contents (atrial fluid and vitreous humor) have anti-inflammatory effects.^[7]^ Uveal melanoma cells may have biological function similar to those normal ocular cells which maintain immune privilege, such as the ability to suppress NK cells through releasing TGF-β or promote T-lymphocyte depletion through the PD-1 pathway, resulting in immune escape.^[8]^ It is the specific ocular immunological environment that makes it rewarding to improve the prognosis and treatments of UM from an immunological point of view.

Over the past decade, long noncoding RNAs (lncRNAs) have gradually emerged as a worldwide scientific hotspot. It has been reported that lncRNAs participate in certain critical biological processes, such as the regulation of proliferation, apoptosis, and metabolism.^[9–11]^ For instance, the interaction of DNA and lncRNA regulates transcription, translation, and epigenetic features.^[12]^ Significantly, some specific immune mechanisms regulated by lncRNAs have been revealed. For example, lncRNAs can enhance the intensity of inflammatory response. Among them, THRIL is an lncRNA that forms a complex with heterogeneous ribonucleoprotein L to bind the TNF-α promoter and induce its transcription.^[13]^

The lncRNA plays multiple roles for UM genesis. Some lncRNAs (i.e., lncRNA NUMB, CANT1, ZNNT1) are therapeutic agents of UM, which can directly act on effector molecules or act as regulatory elements to control transcription and thus activate tumor suppressor pathways or block tumorigenic processes, such as the TGF-β pathway, the JAK2 pathway, and the autophagy pathway.^[14]^ Instead, some lncRNAs are causative agents of UM. For example, the lncRNA P2RX7-V3 variant has been reported to be an oncogene as it is positively associated with the P2RX7 receptor (a ligand-gated ion channel receptor), which leads to tumor cell differentiation, metabolism, migration, invasion, and has been shown to correlate with poor prognosis.^[15]^ More studies have revealed the significance of immune- related lncRNA in the onset and progression of UM. Ding et al found that lncRNA PAUPAR is downregulated in the UM, whose downstream target is HES1 which significantly mediates NOTCH signaling pathway that regulates melanocyte apoptosis.^[16]^ The study of Huang et al reported that low expression of lncRNA PVT1 in UM cells downregulates the expression of EZH2 (a repressor of multiple oncogenes) and results in tumor proliferation and uncontrol apoptosis.^[17]^ Zhou et al suggested that lncRNA GAS5 inhibits epithelial–mesenchymal transition in uveal melanoma by targeting miRNA-21.^[18]^ These studies indicated the enormous potential of these molecules as a tool for UM diagnosis, prognosis and therapy.

Based on the background above, our study aimed at constructing an lncRNA-based prognostic risk model for UM patients. We would perform weighted correlation network analysis (WGCNA) to target immune-related gene clusters. WGCNA does not directly demonstrate the association between thousands of genes and phenotypes, but it is structured on the basis of several gene modules, which may correspond to a few specific biological pathways enabling a data reduction scheme.^[19–22]^ Least Absolute Shrinkage and Selection Operator (LASSO) regression analysis was conducted in order to pinpoint lncRNAs linked to immunity. LASSO is a linear model estimation method to obtain the minimum sum of squared residuals and improve the predictive accuracy and interpretability of regression models.^[23–25]^ Simultaneously, we would implement univariate or multivariate Cox regression analysis which were applied to assess the effect of several variables on survival time and thus were often used in clinical practice for survival analysis.^[26,27]^ The constructed prognosis risk model will categorize samples as groups named high-risk and low-risk. Then we would discover the differential gene expression and mutations between groups to derive the corresponding gene functions through GO analysis and gene set variation analysis (GSVA).^[28,29]^ Finally, we would conduct drug sensitivity studies based on this model to find out its clinical significance. As a supplement, all the statistics analysis process and results visualization would be conducted by R.

## 2. Materials and methods

### 2.1. Acquisition of patient data and extraction of expression matrix

Study samples including RNA transcriptome data and clinical information of UM patients were searched and extracted from TCGA database. The sample included in our study must fulfill the following criteria: i. The patient who has been diagnosed with uveal melanoma based on pathologic results; ii. Complete RNA transcriptional data for the patient are available; iii. Detailed clinical information about the patient must be known, such as TMN stage, survival time, survival status, and drug sensitivity. We retrieved the NCBI database to obtain required genome annotation files (i.e., GRCh38.p13), then extracted the lncRNA and mRNA expression matrices from raw RNA transcriptome data. We downloaded the file of all immune-related genes from ImmPort database, and subsequently retrieved mRNA expression matrices of all immune-related genes from the raw data. This process was accomplished by limma package for R.

### 2.2. Construction of weighted co-expression network and identification of significant gene modules

The limma package and WGCNA package for R were invoked to conduct the WGCNA. The “β” is a soft threshold used to emphasize correlation so that the constructed co-expression network conforms to the standard scale-free network. We invoked the *pickSoftThreshold* function to compute the β. The scale-free *R*^2^ value was set to be 0.90 to get the most suitable β value. Then similar modules were clustered and merged to obtain the significant gene modules. (The minimum number of genes per module was set to 40, and the module dissection threshold was set to 0.3.) Finally, the heatmap was plotted by *R* to represent the correlation between each gene module and clinical features.

### 2.3. Definition of immune-related genes and lncRNAs

The univariate Cox regression analysis was performed by the SURVIVAL package to define the immune-related genes. The genes filtered by *P* value < .001 with confidence interval of 95% were defined as immune-related genes. The forest plot was plotted by *R*.

The Pearson correlation analysis was performed by the limma package. The analysis was conduct to reveal the correlation between the defined immune-related genes and the raw lncRNAs from database in order to define immune-related lncRNAs. The criterion for defining immune-related lncRNA was Pearson correlation coefficient˃0.4 and *P* value < .001. The Sankey diagram to illustrate the correlation was plotted by *R*.

### 2.4. Construction of prognostic risk model by LASSO analysis

LASSO regression analysis was performed by glmnet package to screen the prognostic immune-related lncRNAs. Two immune-related lncRNAs were selected to simulation after sieving. (ZNF667-AS1 and LINC00963). The risk score for an individual sample is then calculated with the crossprod function in *R*. The formula is unified, that is, RiskScore = coef (ZNF667-AS1) × expr (ZNF667-AS1) + coef (LINC00963) × expr (LINC00963). The “coef” is the correlation coefficient while “expr” is the expression of this lncRNA. The training set was divided by median risk score as a criterion into high-risk group and low-risk group. The relevant Kaplan–Meier curves as well as ROC curves were plotted using the survimner package and the timeROC package for R.

### 2.5. Independence verification and nomogram generation

The survival package for R was invoked to conducted univariate/multivariate Cox regression analyses for demonstrating the independence of the model from other indicators. Confidence intervals of 95% and *P* values < .001 were deemed to be statistically significant. The regplot package and the rms package were additionally utilized to plot the nomogram and calibration curves.^[30,31]^

### 2.6. GO analysis and GSVA

One thousand two hundred thirty-one differential genes were filtered out by limma package on the basis of differential expressions between high-risk group and low-risk group (*P*-value < .05, |logFC| > 1). GO enrichment analysis was conducted with clusterProfiler package with corrected *P*-value filtering threshold being .05. The bar plot was generated with ggplot2, and we visualized the result by ggpubr packages.

GSVA was performed by the limma package, GSVA package, and GSEABase package. Immune-related gene sets are available for download from the GSEA website. The pheatmap package was invoked to plot a genetic heatmap to present more centralized immune function.

### 2.7. Genetic mutation analysis

We used the Mafttools package for R to produce waterfall plots in order to display the mutations in groups separately. We computed the tumor mutation burden (TMB) score from patient mutation information previously downloaded from the TCGA database.^[32,33]^ The survival and survminer packages were subsequently invoked to perform 2 survival analyses for TMB and TMB joint risk score. The Kaplan–Meier curves were presented to visualize the results.

### 2.8. Statistics of immunotherapy effectiveness and drug sensitivity

To assess tumor cell evasion to immunotherapy, we utilized the TIDE tool developed by Peng et al to derive immune evasion-related TIDE scores.^[34]^ The violin plots were created while we invoked limma package and ggpubr package to evaluate the correlation between TIDE scores versus risk stage (*P* value < .05).

The half-maximal inhibitory concentration (IC50) was utilized to evaluate the efficacy after medication treatment. We previously downloaded relevant data from the Genomics of Drug Sensitivity in Cancer (GDSC) database and invoked the pRRophetic package to analyze the sensitivity of drug effects for the risk level. We invoked limma package evaluate the correlation between drug sensitivity versus risk stage and graphed box plots by *R*. (*P* value < .05)

## 3. Result

### 3.1. WGCNA and identification of immune-related genes and lncRNAs

**Figure 1.**
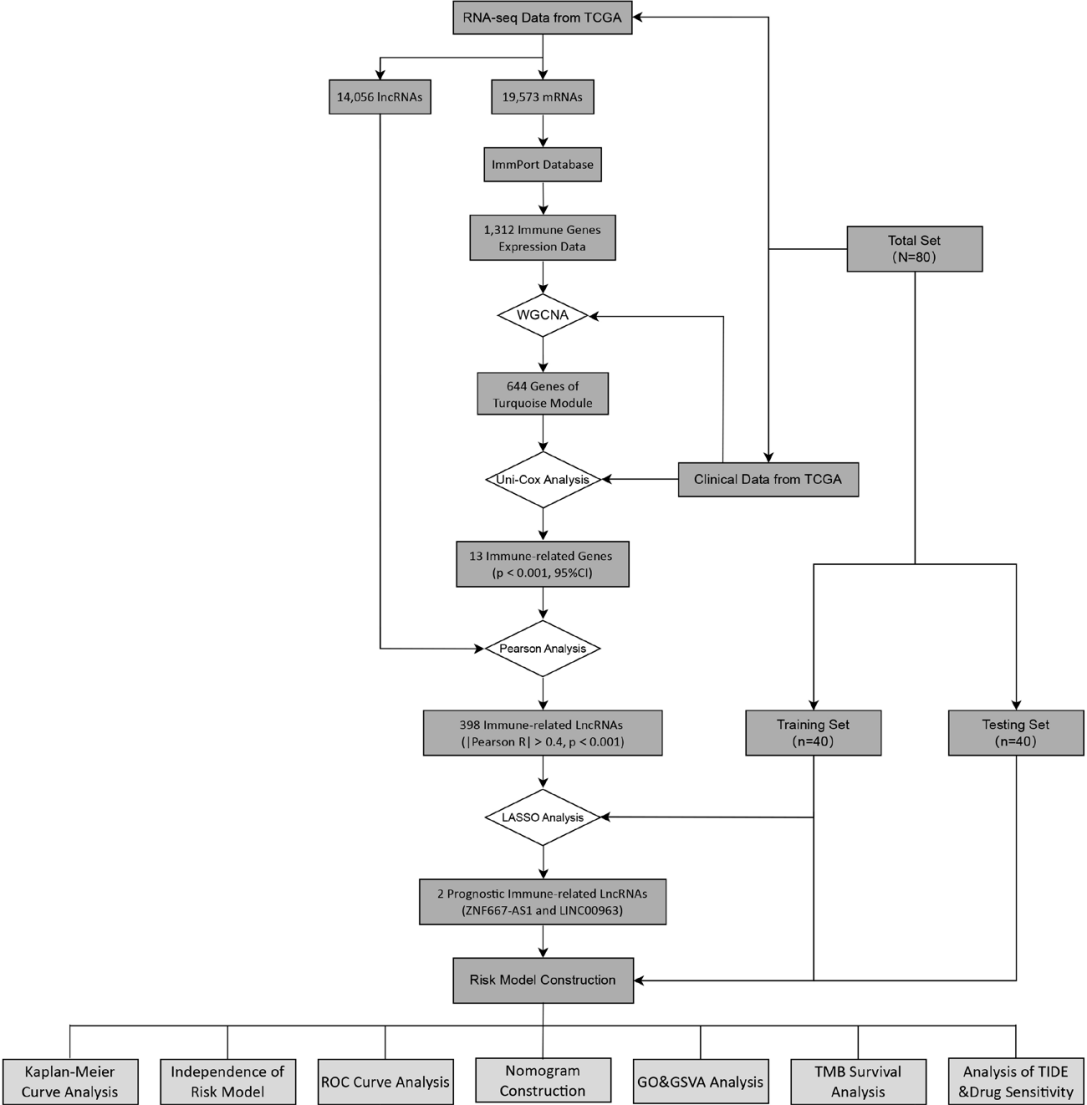
Summarize of the study workflow.

The workflow of data process, model construction, and evaluation of our study is shown in Figure 1.

We retrieved information of 80 UM patients from TCGA database according to the inclusion criteria and extracted their data. Subsequently, 2 expression matrices encompassing 14,056 lncRNAs and 19,573 mRNAs were isolated. The list of all immune genes was obtained from ImmPort database for the final processing to an expression matrix comprising 1312 immune genes of 80 patients.

**Figure 2.**
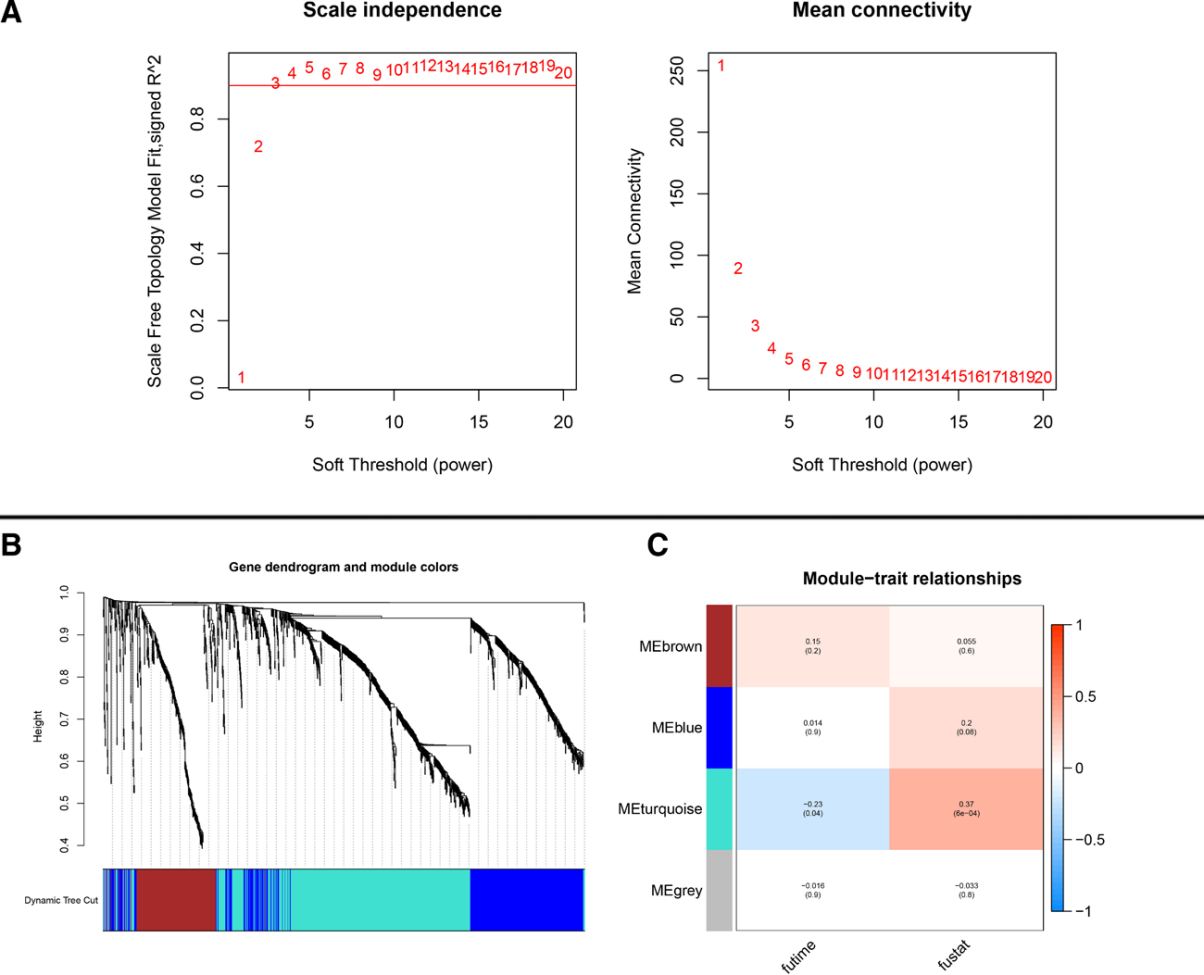
Visualization of WGCNA results. (A) Network topology analysis for various soft-threshold powers. The scale-free *R*^2^ value was set to be 0.90 to get the most suitable β value. β = 3 was selected for subsequent analysis. (B) Gene dendrogram illustrates the gene enrichment of each module; (C) correlation heatmap demonstrates the correlation between each module gene and prognosis. The turquoise module gene correlation is the strongest. (module-futime: correlation coefficient = ‐0.23, *P* value = 0.04; module-fustat: correlation coefficient = 0.37, *P* value = 6e‐04). WGCNA = weighted correlation network analysis.

Weighted correlation network analysis (WGCNA) was next performed to derive co-expressed gene modules from all these immune genes (Fig. 2A). Gene clustering tree diagram demonstrating 1312 immune genes grouped into 4 gene modules (Fig. 2B). Correlations and *P*-values for the 4 gene modules in conjunction with patients’ survival data (vital status and future-time) are shown in Figure 2C. Remarkably, the turquoise module showed the highest correlation and significance with patients’ survival data (module-futime: correlation coefficient = ‐0.23, *P* value = .04; module-fustat: correlation coefficient = 0.37, *P* value = 6e‐04), suggesting the possibility that genes in this module may have critical function in UM, which was featured for our further analysis (see Table S1, Supplemental Digital Content, http://links.lww.com/MD/N530 which shows 644 genes of the turquoise module).

**Figure 3.**
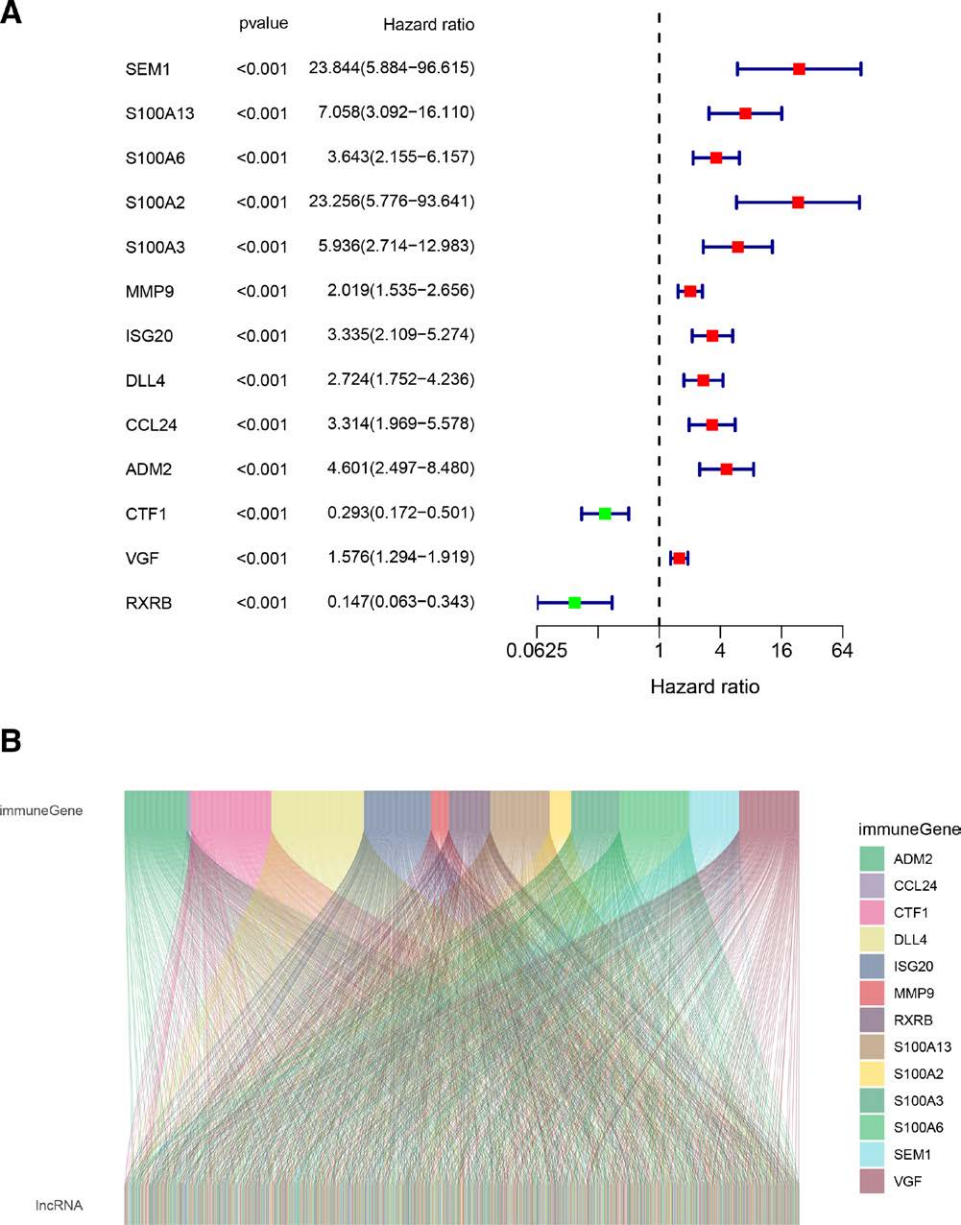
Visualization of immune-related lncRNA screening results. (A) Forest plot of univariate Cox regression analysis results; (B) Sankey diagram shows the interaction of screened lncRNAs with immune genes. lncRNA = long no-coding RNA.

Further, the 13 immune-related genes with strong correlation were concluded by univariate Cox regression analysis. The forest plot as result is presented in Figure 3A. We next conducted Pearson correlation analysis utilizing the limma package for R to identify lncRNAs having high co-expression with these 13 immune genes. The lncRNAs with absolute value of correlation coefficients >0.4 and *P*-values <.001 were identified as immune-related lncRNAs. Ultimately, 398 immune-related lncRNAs were filtered out from 14,056 lncRNAs. The Sankey diagram in Figure 3B illustrates their co-expression relationship (see Table S2, Supplemental Digital Content, http://links.lww.com/MD/N530 which shows 398 immune-related lncRNAs).

### 3.2. Construction of lncRNA-related prognostic model based on LASSO regression

**Figure 4.**
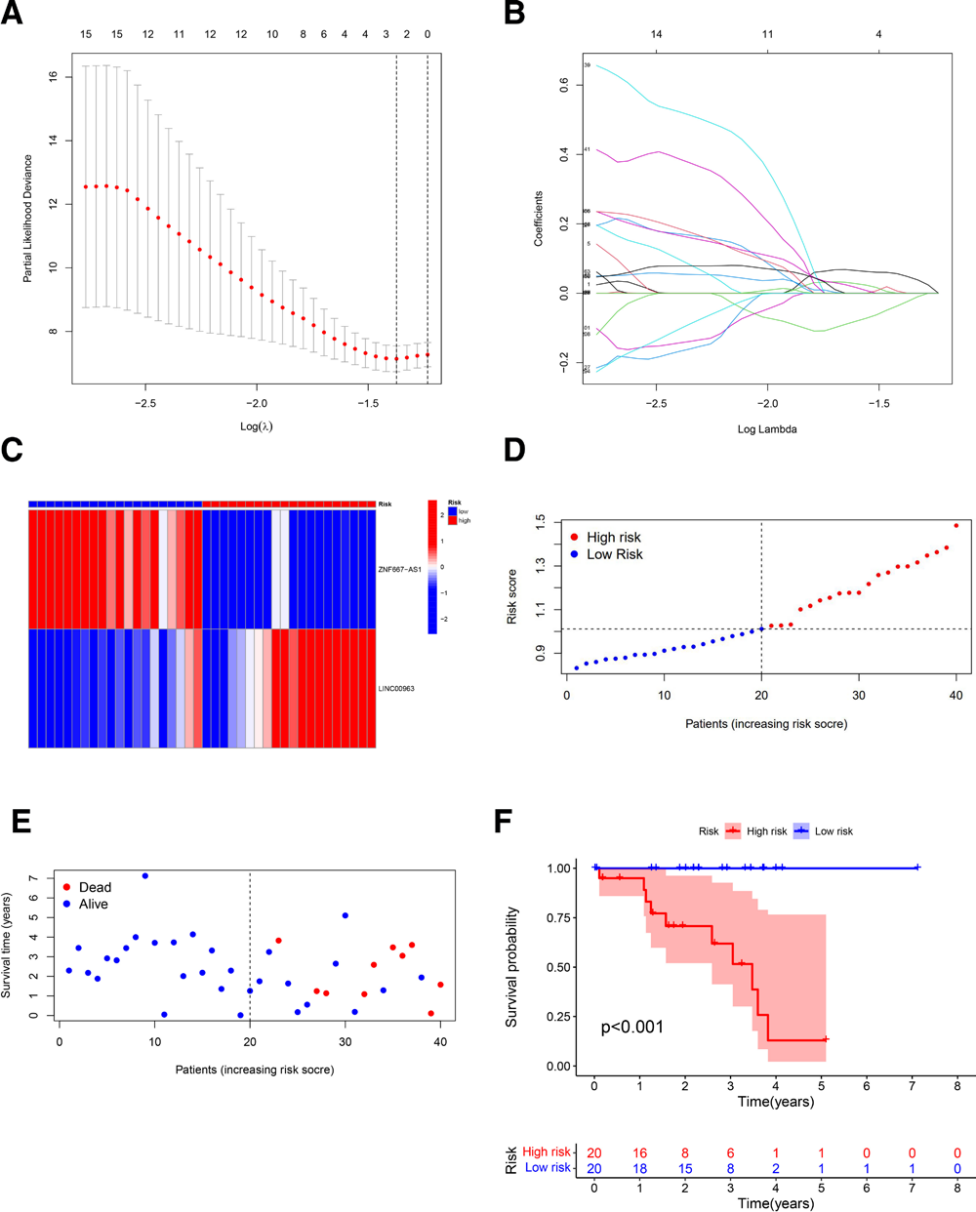
Model construction based on training set patient data. (A and B) Partial likelihood deviance diagram and LASSO coefficient profiles; (C) heatmap of lncRNA expression shows inter-group variation; (D) risk scores of patients in the training set and median-based grouping; (E) scatter plot shows the association between risk stage and survival time; (F) Kaplan–Meier curves reveals prognostic differences between high- and low-risk groups (*P* < .001). LASSO = least absolute shrinkage and selection operator, lncRNA = long no-coding RNA.

**Figure 5.**
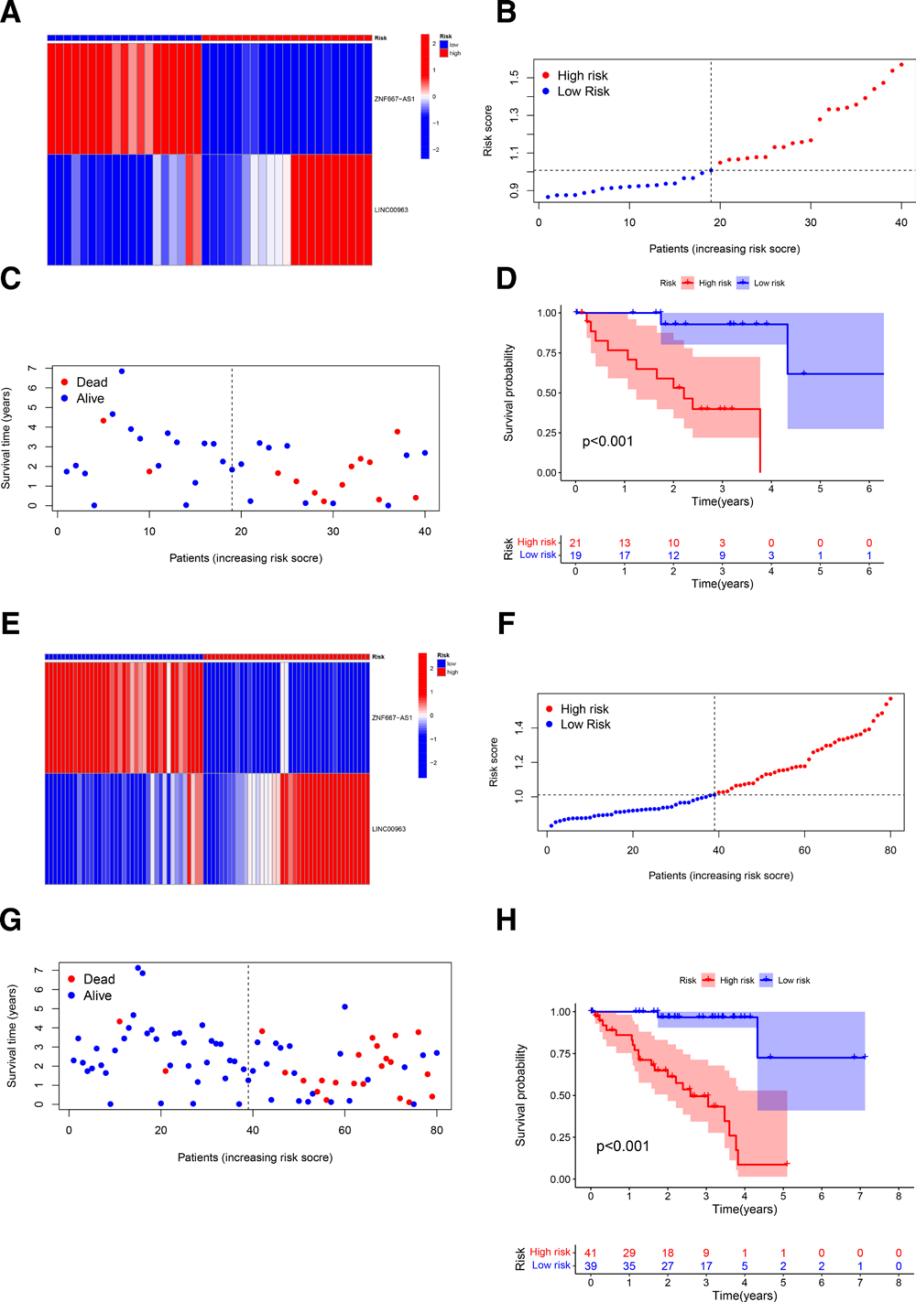
Validation of the model based on testing set and the total set. (A–D) Results of the identical analysis processes based on the testing set; (E–H) results of the identical analysis processes based on the total set.

The total data set including 80 patients is categorized into testing set (n = 40) and training set (n = 40) by the caret package for R. LASSO regression analysis has significant superiority in processing multiple covariance sample data. LASSO analysis was performed by R based on survival data from the training set. The result revealed significant prognostic implications for the expression of 2 immune-related lncRNAs (ZNF667-AS1, coef = ‐0.0237744332866951; LINC00963, coef = 0.0481877269720816). Partial likelihood deviance plot and LASSO coefficient profiles are shown in Figure 4A and B. Next, risk scores were calculated for each sample in the training set according to these 2 lncRNA expressions and correlation coefficients (coef), then we categorized them into a high-risk group (n = 20) or a low-risk group (n = 20) according to the median. Greater risk score indicates the more severe survival risk. Expression heatmap shows visual group variation (Fig. 4C) and risk score curves are illustrated in Figure 4D. Meanwhile, higher risk scores showed poorer prognosis (Fig. 4E). Kaplan–Meier curves visualizes more intuitively the distinction between groups (Fig. 4F). In order to better justify this score-based model, risk scores were computed and grouped in testing set and total set according to the median of the training models with the results exhibiting highly analogous trends. All results above indicate that the higher risk score may imply the worse prognosis (Fig. 5A–H).

### 3.3. Assessment and nomogram generation of lncRNA-related prognostic model

**Figure 6.**
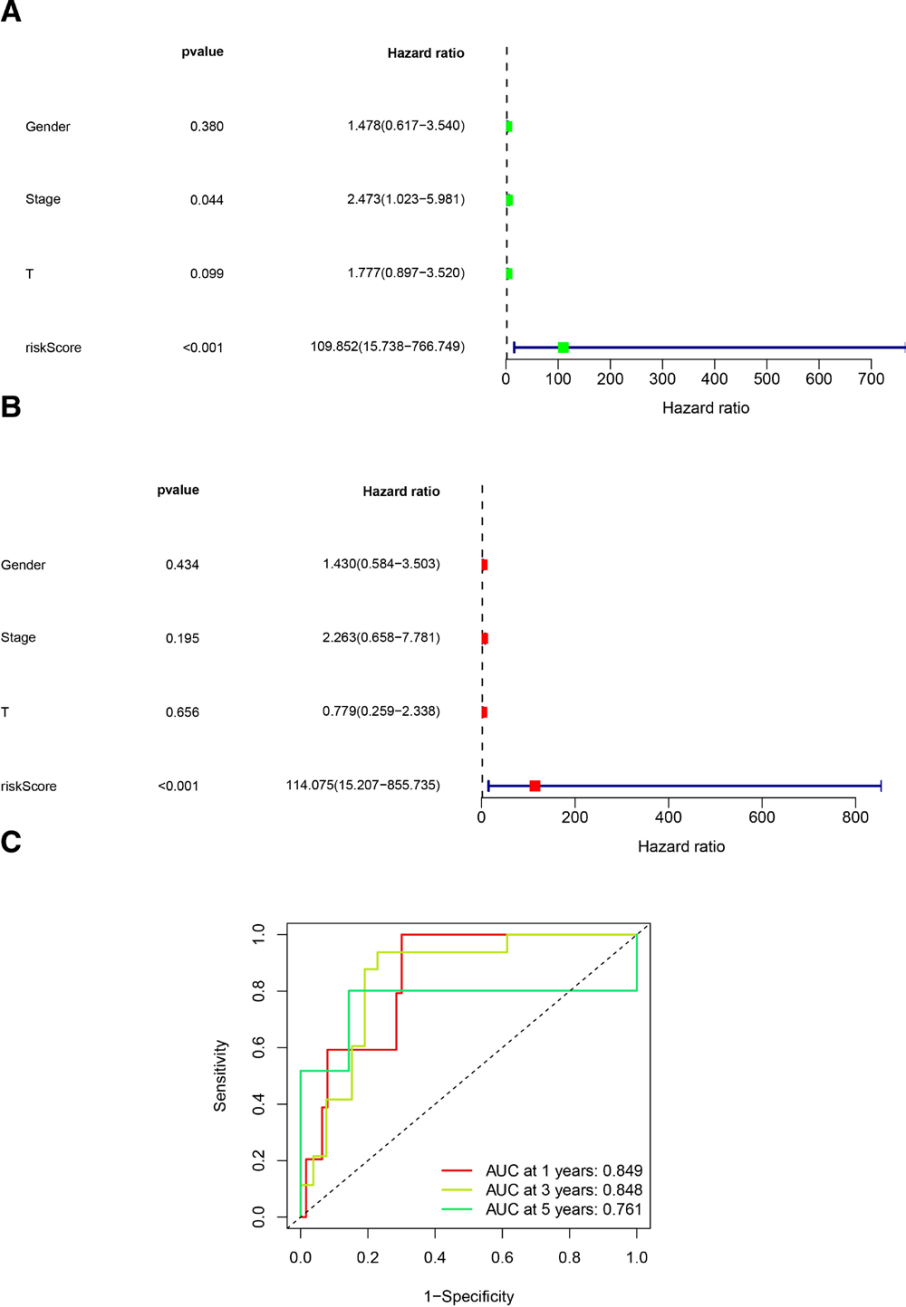
Visualization of evaluation for the predictive capability. (A) The forest plot shows outcomes of univariate Cox regression analysis (hazard ratio = 109.852 (15.738–766.749), *P* value < .001); (B) the forest plot shows outcomes of multivariate Cox regression analysis (hazard ratio = 114.075 (15.207–855.735), *P* value < .001); (C) ROC curves demonstrate the specificity and sensitivity of the model (1-year-AUC = 0.849, 3-year-AUC = 0.848 and 5-year-AUC = 0.761). AUC = area under the curve.

In order to verify the independence of the model, gender, TNM stage(especially T stage of which) and risk scores from the total set (n = 80) were selected to conducted univariate and multivariate Cox regression analyses. The results indicated that the risk score was significantly independent of other clinical parameters in either univariate (hazard ratio = 109.852 (15.738–766.749), *P* value < .001) or multivariate (hazard ratio = 114.075 (15.207–855.735), *P* value < .001) analyses (Fig. 6A and B).

The ROC curve is frequently used to reflect the association between sensitivity and specificity of a prediction model. The value of the area under the curve (AUC) ranging from 0 to 1 is positively correlated with the prediction accuracy. Figure 6C demonstrates the ROC curve of this lncRNA-related model, implying high predictive accuracy for 1-year, 3-year, and 5-year survival (1-year AUC = 0.849, 3-years AUC = 0.848, and 5-years AUC = 0.761).

**Figure 7.**
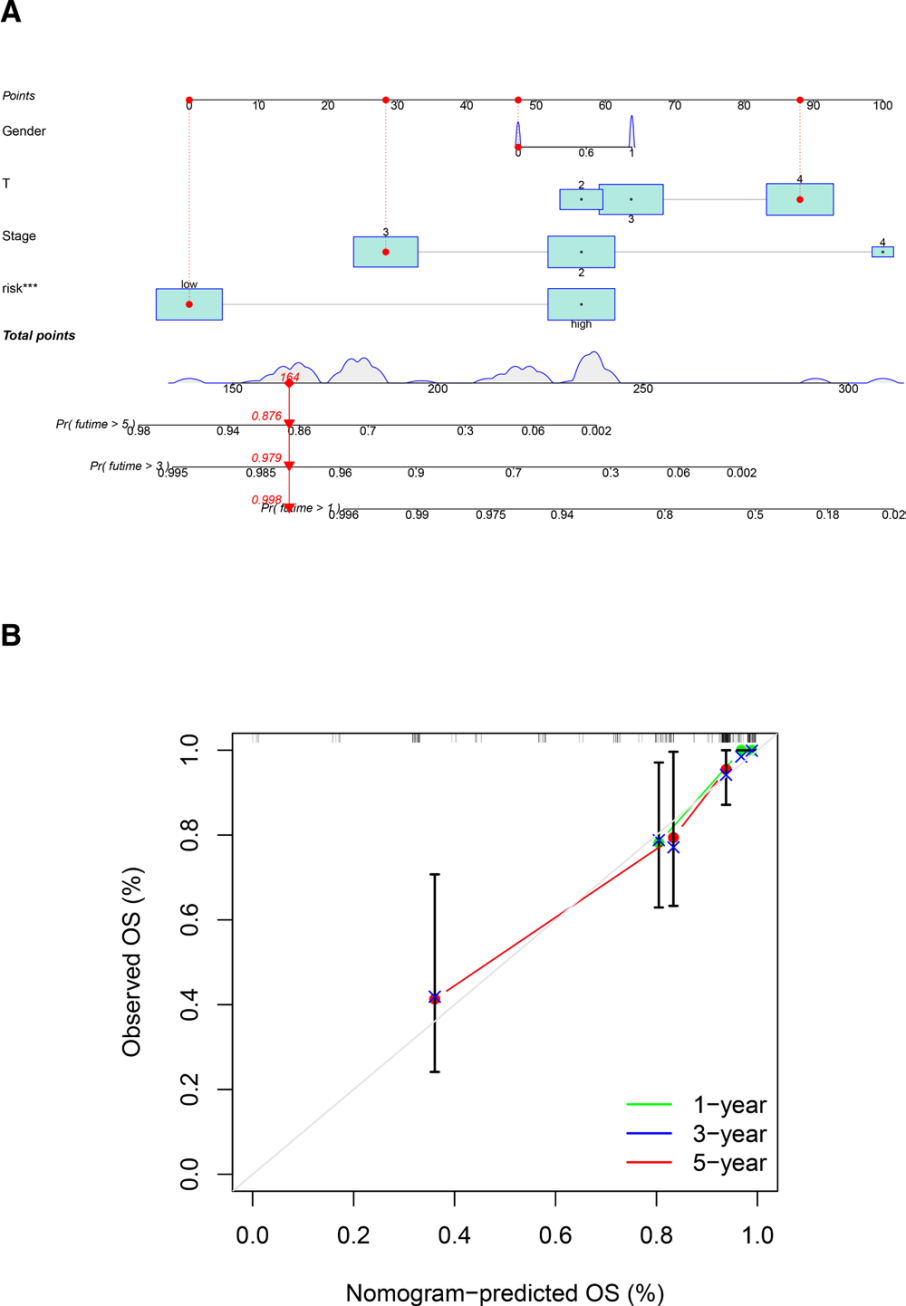
Nomogram construction and assessment. (A) The Nomogram with parameters consisting of risk score, risk stage and other clinical characteristics; (B) calibration graph shows the consistency of nomogram predicted values and actual observed values.

Nomogram is based on a multivariate regression analysis that integrates multiple indicators, using scaled lines to represent the interrelationships between the variates, with the evaluation of the forecast values in the form of assigned scores. Nomogram shows that risk stage (high or low) has a dominant position in terms of prediction accuracy compared with other clinical indicators (Fig. 7A). The accuracy of this nomogram was evaluated in the calibration plot and the diagram revealed the model has high similarity between observed and predicted values (Fig. 7B).

### 3.4. Genetic variation between groups and related functional enrichment analysis

**Figure 8.**
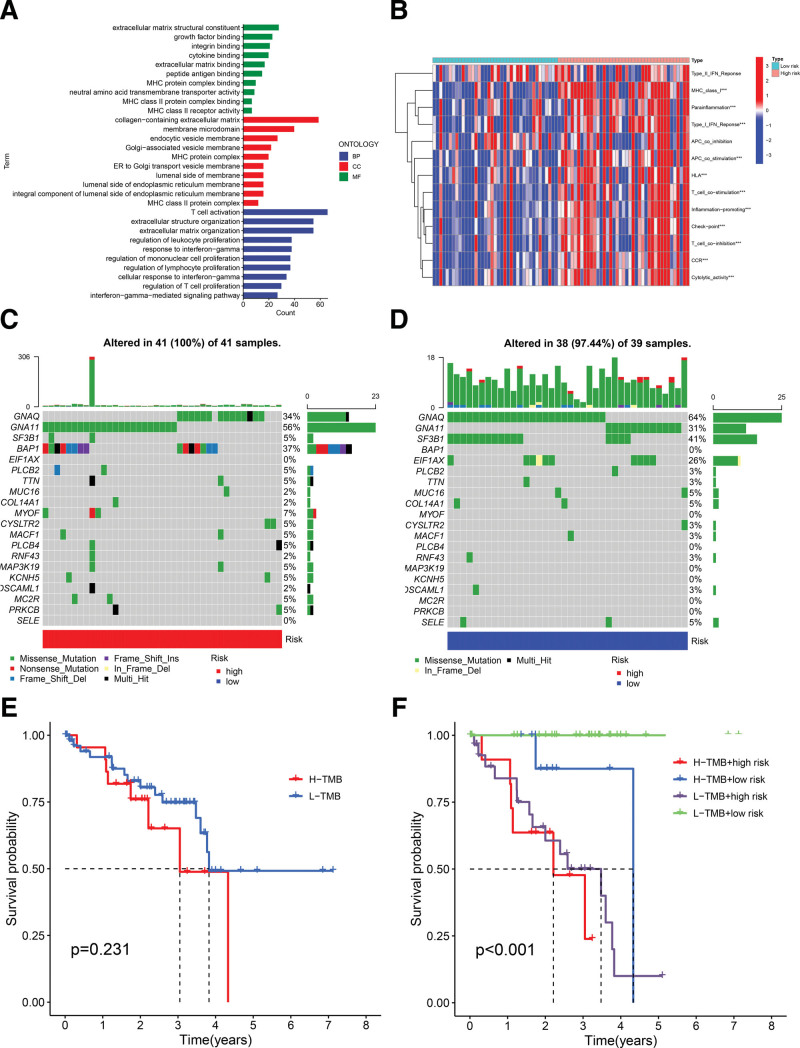
Visualization of gene enrichment analysis and inter-group mutational variation. (A) The histogram shows the results of GO enrichment analysis, indicating that these differential genes are closely associated with immune function; (B) the heatmap visualizes GSVA results; (C and D) waterfall plots demonstrate the mutations from the 2 groups, revealing significant differences concerning 2 genes (GNAQ and GNA11); (E) Kaplan–Meier curves indicate poor prognostic relevance based on TMB scores; (*P* = .231); (F) Kaplan–Meier curves show curve better prognostic capability when involving the risk stage. (*P* < .001). GSVA = gene set variation analysis, TMB = tumor mutation burden.

Analysis of differential gene expression between patients in the low-risk (n = 39) group versus high-risk group (n = 41) was conducted by limma package for R. A total of 1231 differential genes were filtered out (*P* value < .05, |logFC| > 1). GO analysis was conducted on these genes and the bar plot is presented in Figure 8A. Interestingly, the results revealed that these differential genes might regulate biological processes such as the generation of collagen-containing extracellular matrix and the T cell activation and concentration in a series of immune response processes involving lymphocytes. GSVA analysis was also conducted with the immune function-related gene set to investigate the distinct biological processes between groups. Likewise, the heat map displays that the intergroup variation is intimately related to immune functions (Fig. 8B).

Mutation information of these patients was extracted from TCGA database to analyze the accumulation of gene mutations. Waterfall plots visualizes gene mutations of each group (Fig. 8C and D). Interestingly, there is a significant distinction between groups in the mutation status of the 2 genes GNAQ and GNA11 which are already proven to be associated with UM. Hence, it strongly demonstrates the reliability of this model.

Based on these mutation data, the TMB score was also computed for every sample. The median TMB score was the criterion to categorize the sample into the high mutation group or the low mutation group. Higher TMB scores indicate greater mutational load, which is usually positively associated with a worse prognosis for the cancer.^[34]^ However, there was no significant prognostic difference in grouping according to TMB scores.(*P* value = .231) Possible reasons for this result is that UM has excessive mutation sites or mutated genes, which leads to a insignificant mutation load variation (Fig. 8E). Interestingly, after incorporating the risk stage based on our study, the curves show a conspicuous discrepancy (*P* value < .001) (Fig. 8F). Remarkably, there is no visible difference between H-TMB/H-risk group versus L-TMB/H-risk group while revealing tangible distinction versus H-TMB/L-risk group. It suggests that this lncRNA-related model has greater prognostic relevance over TMB scores.

### 3.5. Implication of lncRNA-related prognostic model for immunotherapy strategies and drug sensitivity

**Figure 9.**
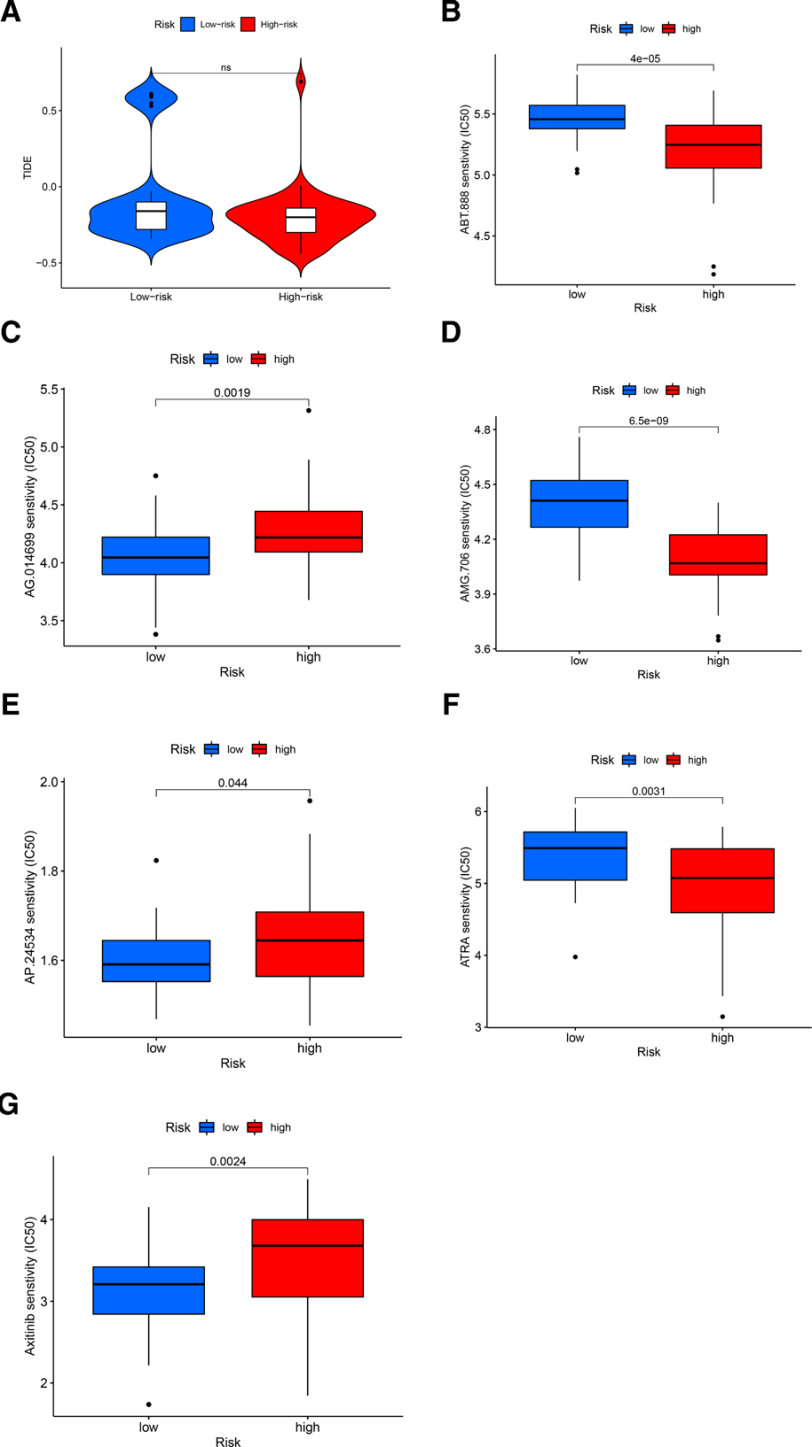
Visualization of sensitivity of the model to immunotherapy and related specific drugs (*P* < .05). (A) The violin plot shows high-risk group is more sensitive to immuno-infiltrative therapy; (B) the box plot shows ABT.888 sensitivity to risk stage; (*P* value = 4e‐05); (C) the box plot shows AG.014699 sensitivity to risk stage; (*P* value = 0.0019); (D) the box plot shows AMG.706 sensitivity to risk stage; (*P* value = 6.5e‐09); (E) the box plot shows AP.24534 sensitivity to risk stage; (*P* value = .044); (F) the box plot shows ATRA sensitivity to risk stage; (*P* value = .0031); (G) the box plot shows Axitinib sensitivity to risk stage (*P* value = .0024).

The TIDE algorithm can be applied to forecast response to immunotherapy.^[35]^ Figure 9A shows that the high-risk group responded to immunotherapy more sensitively than the low-risk group, suggesting that our model has potential to facilitate the selection of immunotherapy strategies. We analyzed the sensitivity of the model to drug treatment by extracting IC50 data from the GDSC database for the drug of interest. Box plots depict 6 drugs with high sensitivity to this model (Fig. 9B–G). Notably, the sensitivity of some drugs showed anomalous performance between the low-risk and high-risk groups. For example, the high-risk group has higher sensitivity to AG-014699 (Rucaparib) which is commonly used with advanced ovarian cancer combined with BRCA mutations.^[36]^ This suggests that our mechanistic studies of UM could start from common mutation sites in ovarian cancer and also points to possible directions for future clinical cohort studies.

## 4. Discussion

A problem that has long plagued ophthalmologists is that UM is a malignant tumor with a negative long-term prognosis.^[37]^ More than 50% of patients will develop metastases, with common sites including the liver and lungs, suggesting the alarming lethality of UM.^[38,39]^ Some studies have revealed some morphological prognostic indicators of tumor metastasis or death, including tumor basal diameter, tumor thickness, and the occurrence of intraocular hemorrhage and extraocular extension.^[40]^ At present, the more authoritative clinical staging system is the TNM grading which combines with tumor size, regional lymph nodes and metastasis.^[41]^ However, these prognostic tools are often not adapted to clinical needs because the accuracy and correlation are not significant enough. In our study, we developed an immune-related lncRNA-based risk prognostic model and evaluated its accuracy and sensitivity.

In this study, we focused on utilizing lncRNA to predict the prognosis of a tumor, namely uveal melanoma. Recently, it has been demonstrated that inextricable links between lncRNAs and tumorigenesis or progression exist. The functions of lncRNA can be succinctly summarized into 4 dimensions: signaling, decoy, guidance and structure.^[42]^ For instance, lncRNAs directly regulate signal pathways of tumor suppression. MEG3, as lncRNA, specifically targets genes involving the TGF-β pathway by binding to the distal regulatory region of the promoter and possesses the property of activating p53 transcription.^[43,44]^ Several studies have pointed out the potential of lncRNAs as biomarkers for cancer diagnosis, such as the high expression of PCAT-1, PRNCR1, PCGEM, PlncRNA1, and PCAT-18 in prostate tumors.^[45]^ Although there have been studies demonstrating the correlation between lncRNA differential expression and UM progression, the precise intracellular mechanisms remain to be elucidated.^[46,47]^

In our study, a cluster of genes closely related to immune function was first filtered out from 1312 immune-genes based on gene expression information and survival time from 80 patients, which evidently improved the target-orient compared with other previous studies. After LASSO analysis, the construction of our risk model was predicated on these 2 lncRNAs, ZNF667-AS1 and LINC00963. A recent study has claimed that Zinc finger protein 667-antisense RNA 1 (ZNF667-AS1) might be positively correlated with MEGF10, both of which are associated with proliferation suppression and apoptosis induction, presenting a significant downregulation of expression in UM cells.^[48]^ In similar studies of colorectal and cervical cancers, it was also reported that the decreased level of ZNF667-AS1 reduced the inhibitory effect on tumor proliferation and metastasis.^[49,50]^ This negative modulation is consistent with the results of the correlation coefficients calculated in our study (coef = ‐0.0237744332866951). Concurrently, LINC00963 has been proven to involve in cancer progression. Some antisense oligonucleotides can specifically complement with microRNAs and inhibit their functions, termed as “microRNA sponges.”^[51]^ It has been reported that LINC00963, as lncRNA, act as such spongy interactions in cancer development, affecting specific miRNA-mediated axis to exert a regulatory role.^[52–56]^ However, there has been no reports revealing the function of LINC00963 in uveal melanoma until now, which imply the further research.

According to this risk model, we analyzed mutations that varied in groups. Interestingly, we discovered significant mutation differences of 2 genes, GNAQ and GNA11. The robust relationship between these mutations versus the occurrence of UM has been reported.^[4]^ GNAQ and GNA11 respectively encode 2 proteins (Gαq and Gα11), which belonging to the G protein alpha subunit family.^[57]^ Q209L mutations in GNAQ or GNA11 were detected in approximately 80% of UM patients, which may persistently activate the G protein signaling pathway.^[58]^ Gαq has been reported to provoke activation of the FAK/Hippo-YAP pathway, which mediates uncontrolled proliferation and carcinogenesis in turn.^[59]^ Hence, these pathways indicate a potential therapeutic direction for uveal melanoma.

We also demonstrated the superiority of this lncRNA prognostic model compared to other tumor prognostic tools. The TMB score has been recognized as a popular tumor prognostic tool in recent years.^[60]^ According to data from 80 patients, our study presented that the immune-lncRNA related model has a more significant prognostic value than the TMB score in terms of accuracy and sensitivity. Also, we introduced the TIDE tool to assess the relevance of this model on immunotherapy escape and obtained convincing results. Finally, we proposed 6 drugs that are sensitive to the model based on the GDSC database (i.e., ABT.888, AMG.706, AG.014699, AP.24534, ATRA, and Axitinib) This indicates that the prognostic model we constructed has remarkable clinical treatment directives. Interestingly, we found Rucaparib (PARP inhibitor) as a more effective drug for high-risk UM patients, which may provide a new option for future UM medications.

In addition, how to integrate this risk model into clinical applications is a critical question. The lncRNA is a prominent direction for clinical diagnostic applications due to its highly variable expression between different cells. For example, urine lncRNA PCA3 is already a biomarker for early prostate cancer, with test strips being tested in clinical trials. Because of the high accessibility of urine, the PCA3-strip may become an important aid in the diagnosis of prostate cancer in the future.^[61]^ For UM, current diagnosis mainly depends on fundoscopy, ultrasonography, fundus fluorescence angiography and CT. The fine needle aspiration biopsy (FNAB) is usually not the preferred option for its invasiveness. However, FNAB is a necessary operation for patients with atypical clinical presentation, dense cloudy media, possible uveal metastasis from the primary tumor, and for who require histopathologic confirmation prior before enucleation.^[62]^ Our risk model combined with FNAB allows for a better assessment of patient prognostic risk. Ophthalmologists or pathologists can sequence lncRNA from FNAB samples and group them based on the risk model, thereby making better clinical decision of eyeball enucleation, localized patch radiation therapy (LPRT) or medication.

However, the study still has a long way to go. The samples in this study were all from the TCGA database, which may have resulted in statistical bias. Future studies should focus on the generalizability of our findings to other databases. Since UM patient data are scarce in the TCGA database compared to other tumors, we had to retrieve information for only 80 patients. Subsequent studies will aim to seek more samples and focus on other lncRNAs related to biological function with the purpose of improving the prognostic model. Moreover, subsequent diagnostic cohort studies based on this model could be conducted to explore the diagnosis implications and therapeutic cohort study could be conducted to validate the model’s guidance on the drug treatment.

## 5. Conclusion

Our study took the immune-functional gene cluster as the entry point, derived immune-related lncRNAs through calculation, successfully finished the construction and evaluation towards this prognostic risk models. This risk model combined with FNAB offers a better criterion for UM diagnosis and therapy. Our future study will focus on expanding the applicability of the model, as well as conducting clinically relevant cohort studies.

## Author contributions

**Conceptualization:** Nengqi Lin, Dongliang Yang, Wei Liu.

**Data curation:** Nengqi Lin.

**Formal analysis:** Nengqi Lin.

**Funding acquisition:** Wei Liu.

**Investigation:** Nengqi Lin.

**Methodology:** Nengqi Lin.

**Project administration:** Nengqi Lin.

**Resources:** Nengqi Lin.

**Software:** Nengqi Lin.

**Supervision:** Nengqi Lin.

**Validation:** Nengqi Lin.

**Visualization:** Nengqi Lin.

**Writing – original draft:** Nengqi Lin, Ruohan Lv.

**Writing – review & editing:** Nengqi Lin, Ruohan Lv.

## Supplementary Material


